# Subject-Specific Finite Element Analysis of the Human Femur Using Radiation-Free Three-Dimensional Zero-Echo-Time Magnetic Resonance Imaging (3D ZTE MRI): A Feasibility Study

**DOI:** 10.3390/life16071168

**Published:** 2026-07-14

**Authors:** Ann-Kristin Becker, Bastian Klaan, Iman Soodmand, Chris Lappe, Daniel Cantré, Michael Dau, Marc-André Weber, Janos Zierath, Rainer Bader, Jan-Oliver Sass, Maeruan Kebbach

**Affiliations:** 1 Research Laboratory for Biomechanics and Implant Technology, Department of Orthopedics, Rostock University Medical Center, Doberaner Straße 142, 18057 Rostock, Germany; iman.soodmand@med.uni-rostock.de (I.S.); or rainer.bader@uni-rostock.de (R.B.); maeruan.kebbach@med.uni-rostock.de (M.K.); 2 Institute and Policlinic of Radiology, Pediatric Radiology and Neuroradiology, Rostock University Medical Center, 18057 Rostock, Germany; bastian.klaan@med.uni-rostock.de (B.K.); chris.lappe@med.uni-rostock.de (C.L.); daniel.cantre@med.uni-rostock.de (D.C.); marc-andre.weber@med.uni-rostock.de (M.-A.W.); 3 Department of Oral, Maxillofacial Plastic Surgery, Rostock University Medical Center, 18057 Rostock, Germany; michael.dau@med.uni-rostock.de; 4 Technical Dynamics, Faculty of Mechanical Engineering and Marine Technology, University of Rostock, 18051 Rostock, Germany; janos.zierath@uni-rostock.de; 5 Department Life, Light & Matter (LL&M), University of Rostock, 18059 Rostock, Germany

**Keywords:** zero-echo-time, magnetic resonance imaging, CT-like MRI, finite element analysis, femoral bone, material properties

## Abstract

Finite element (FE) modeling is widely used in biomechanical research, enabling computational investigations of anatomical structures. To develop accurate FE models, high-resolution medical imaging such as computed tomography (CT) is essential. However, CT exposes patients to ionizing radiation. Recently, radiation-free three-dimensional zero-echo-time magnetic resonance imaging (3D ZTE MRI) has shown promising results for bone visualization. Therefore, this feasibility study investigated whether 3D ZTE MRI is reliable for creating subject-specific FE models. Human femora from four female subjects were imaged using 3D ZTE MRI. Static FE analyses of the femora were performed in Abaqus/CAE, assuming a biphasic homogenous linear-elastic material. A sensitivity analysis was conducted to evaluate varying bone material properties. After loading with 2000 N, the mean displacement of the femoral head amounted to −1.30 ± 0.26 mm (vertical) and −9.03 ± 2.21 mm (horizontal), while the femoral neck exhibited compressive (inferior: −1366.03 ± 182.70 µm/m) and tensile (superior: 1038.69 ± 135.82 µm/m) strains. Overall, the results demonstrate displacement and strain predictions similar to previous experimental and computational studies based on CT data. Therefore, despite several simplifications, these findings confirm the feasibility of radiation-free 3D ZTE MRI for subject-specific FE modeling, enabling future simultaneous assessment of bone morphology and surrounding soft tissues.

## 1. Introduction

Finite element (FE) models are frequently used in biomechanical research to support pre-clinical evaluation or clinical decision-making [1,2,3] and enable detailed simulations of the behavior of anatomical structures under various physiological and pathological conditions [4]. For instance, they are commonly applied to assess fracture risk, optimize surgical interventions, and evaluate biomechanical function [5]. Their ability to analyze complex geometry and material properties makes them of great interest for individualized clinical applications and subject-specific modeling approaches [6].

To build accurate, subject-specific FE models, high-resolution medical imaging is essential. In this regard, computed tomography (CT) is the most commonly used imaging technique for bones due to its high resolution and other advantages, such as a reliable and validated correlation between Hounsfield units and bone mineral density, which allows the assignment of subject-specific, heterogeneous bone material properties [7,8,9,10]. However, CT imaging exposes patients to ionizing radiation, which is associated with a non-negligible health risk and leads to a higher cancer risk [11]. In this context, Smith-Bindman et al. [12] projected, in a model-based risk analysis, that if current practices persist, CT-related radiation could account for approximately 5% of all new cancer diagnoses annually in the USA. Other studies project that one in 270–600 people who underwent CT coronary angiography at age 40 years will develop cancer as a result of that scan [12]. These findings underscore the need for reliable, radiation-free alternatives for imaging in musculoskeletal research that still provide sufficient image resolution, e.g., for subject-specific FE modeling.

In recent years, other imaging approaches such as optical 3D scans [13] and three-dimensional (3D) magnetic resonance imaging (MRI) techniques capable of generating CT-like bone contrast for direct visualization of osseous structures have gained increasing attention [14,15,16,17,18,19,20]. However, conventional MRI sequences are limited in their ability to visualize bone due to the very short T2 and T2* relaxation times and low proton density. This causes rapid signal decay and a minimal detectable signal [14,15], e.g., making them unsuitable for FE modeling of bone structures. To overcome these limitations, MRI sequences such as ultrashort echo time (UTE) and zero-echo-time (ZTE) have been developed to capture signal from short-T2 tissues by minimizing the delay between radiofrequency excitation and signal acquisition [21]. The difference between the two sequences lies in the application time of the readout gradient [22]. Images obtained with UTE and ZTE sequences achieve echo times in the range of tens of microseconds, enabling the detection of cortical bone signal before substantial decay occurs. As a result, UTE and ZTE MRI can generate images with bone contrast and anatomical detail that approach those provided by CT while avoiding exposure to ionizing radiation [17,21,23,24]. These techniques enable the assessment of bone and adjacent soft tissues such as muscles, ligaments and menisci, within a single MRI examination and potentially reduce the need for multimodality imaging in musculoskeletal applications [17,24,25,26,27,28]. Despite these advantages, current limitations include sensitivity to off-resonance effects, image blurring at air–bone interfaces, and the requirement for dedicated hardware, sequence optimization, and post-processing techniques to achieve optimal image quality [14,26]. Although UTE and ZTE MRI have demonstrated promising capabilities for bone visualization, their suitability for generating subject-specific FE models remains insufficiently investigated.

Therefore, this feasibility study aimed to investigate whether 3D ZTE MRI can serve as a suitable radiation-free alternative to CT for generating subject-specific FE models for biomechanical research.

For that, the human femoral bone was selected as a representative anatomical case. The femur plays a central role in locomotion and load transfer within the musculoskeletal system [29]. It is also among the most frequently studied bones in biomechanics using FE analysis [3,30]. In clinical practice, CT imaging of the femur is commonly performed for trauma assessment, total hip arthroplasty planning, and evaluation of osteoporosis [31,32]. Consequently, the femur has become a standard reference structure for FE analyses in musculoskeletal research [32]. Previous studies have also demonstrated its suitability for benchmarking computational modeling approaches [8,9]. To assess the feasibility of using radiation-free 3D ZTE MRI for subject-specific FE modeling, a workflow including image acquisition, segmentation, post-processing, and FE model generation was developed to investigate displacements and surface strains. We hypothesized that the resulting FE models would produce biomechanically plausible results comparable to those reported in the literature [9,33,34,35,36,37,38,39].

## 2. Materials and Methods

**Figure 1:**
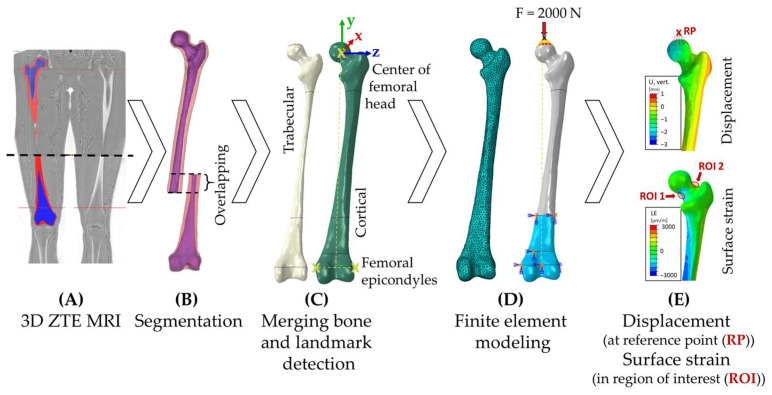
Workflow of the proposed feasibility study. (**A**) Medical imaging with CT-like 3D ZTE MRI in two overlapping sequences shown by the black dashed line, (**B**) segmentation of the human femoral bone and separation into cortical and trabecular bone, (**C**) merging both bone parts and detection of landmarks, (**D**) finite element modeling, (**E**) biomechanical analysis of vertical and horizontal displacement at the reference point (RP) of the femoral head and logarithmic surface strain (LE) in the superior and inferior region of interest (ROI) at the femoral neck.

This feasibility study was designed to evaluate the use of CT-like 3D ZTE MRI to generate subject-specific FE models of the human femur bone for biomechanical applications. For this, the methodological workflow shown in Figure 1 comprised 3D ZTE MRI-based image acquisition (Figure 1A), semi-automated segmentation (Figure 1B), geometric post-processing (Figure 1C), and FE model generation (Figure 1D) to create subject-specific femoral FE models. Using this workflow, we conducted biomechanical analysis and studied the vertical and horizontal displacements of the femoral head and the surface strains at the superior and inferior femoral neck regions (Figure 1E). The results should be interpreted alongside the computational round-robin study with experimental validation by Kluess et al. [9] and other literature [33,34,35,36,37,38,39] to assess the feasibility of using radiation-free 3D ZTE MRI for subject-specific FE modeling.

### 2.1. Participant Recruitment and Data Acquisition

Four female participants (age: 30.4 ± 4.6 years; BMI: 22.2 ± 1.5 kg/m^2^, see Table 1 for more information) were recruited as part of a broader research project (project sponsor Federal Ministry for Economic Affairs and Energy (BMWE) from the “Zentralen Innovationsprogramm Mittelstand” (ZIM); ZIM network funding: VDI/VDE Innovation + Technik GmbH, grant number: 16KN081073) that required a homogeneous cohort, and imaging data were shared across multiple studies. Written informed consent was obtained from all participants before data collection. The study was conducted in accordance with the approved ethics protocol (approval number A 2024-0025) by the local ethics committee of the Rostock University Medical Center. Inclusion was limited to healthy adults, while participants were excluded if they presented with any of the following conditions: (1) joint replacement including temporomandibular, shoulder, hip, knee, or ankle joint; (2) acute injuries or surgical interventions of the lower extremities and/or spine within the last 12 months; and (3) joint, muscular, or systemic diseases, e.g., multiple sclerosis, rheumatic diseases, osteoporosis, or chronic obstructive pulmonary disease.

### 2.2. Medical Imaging

All images were acquired using a clinical whole-body 3.0-tesla MRI scanner (GE Premier, GE HealthCare, Chicago, IL, USA). All subjects were positioned supine, and the right leg was fixed to avoid motion-induced artifacts. The imaging of the lower extremity was performed using a combination of 30-channel and 21-channel receive flex coils and a 60-channel receive spine coil (Figure 1). Two overlapping bilateral isotropic coronal 3D ZTE sequences (oZTEo) were acquired, covering the entire femoral bone. The total imaging time was 2 × 272 s = 544 s. Detailed imaging parameters are provided in Table 2.

### 2.3. Segmentation and Surface Processing of Femoral Bone Geometries

For computational analysis, the geometries of the right femoral bone were reconstructed as 3D models based on the approach described by Kluess et al. [9]. Image data obtained by 3D ZTE MRI were processed in MIMICS 25.0 (Materialise NV, Leuven, Belgium). Since 3D ZTE MRI grayscale values do not correspond to Hounsfield Units and no validated phantom-based calibration currently exists for this modality, the bone was first segmented as a whole using semi-automatic image-intensity thresholding. Subsequently, the trabecular compartment was segmented and subtracted to obtain the cortical bone structure (Figure 1). Subsequently, a reconstruction was conducted; the resulting surface models in STL format were processed in Geomagic Studio 2013 (Geomagic Inc., Morrisville, NC, USA) and converted into volume models (non-uniform B-spline surfaces). This process included merging the proximal and distal femoral segments as well as smoothing operations to reduce stair-step artifacts on the bone surface and to improve subsequent mesh quality. Additionally, the medial and lateral femoral epicondyles and the center of the femoral head were identified, with the latter determined using a best-fit sphere approach [10].

### 2.4. Finite Element Analysis

The 3D geometries of the trabecular and cortical parts of the four femoral bones were imported into the FE software Abaqus/CAE v.2022 (Dassault Systèmes Simulia Corp, Vélizy-Villacoublay, France), and the static-implicit solver was used to perform the analysis of the models. The modeling approach was based on the protocol described by Kluess et al. [9], who conducted computational studies based on CT data as well as experimental studies, making it the primary reference for evaluating the feasibility of using radiation-free 3D ZTE MRI for subject-specific FE models.

**Figure 2:**
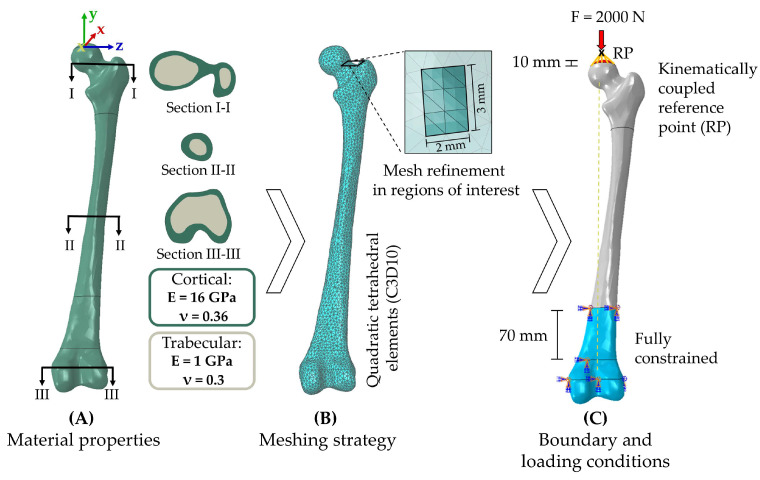
Finite element (FE) modeling of the human femoral bone, including (**A**) cortical and trabecular material properties, (**B**) meshing with quadratic tetrahedral elements (C3D10) and mesh refinement around regions of interest representing strain gauge locations, and (**C**) boundary and loading conditions of the FE analysis, with the distal part of the femur constrained and a 2000 N load applied along the femoral load axis [40] according to Kluess et al. [9].

Firstly, a femoral coordinate system was defined according to Wu et al. [40], with the origin located at the center of the femoral head (Figure 1). The y-axis was oriented cranially from the midpoint of the femoral epicondyles to the origin; the z-axis lay in the epicondylar plane and pointed laterally; and the x-axis was defined perpendicular to both, pointing anteriorly. Two 3 × 2 mm^2^ rectangle areas were defined as regions of interest (ROI) on the inferior (ROI 1) and superior (ROI 2) sides of the femoral neck (Figure 1) to represent virtual strain gauges on the bone surface. Their anatomical locations were defined according to the reference study by Kluess et al. [9]. Trabecular and cortical geometries were merged, and linear-elastic and isotropic material properties were assigned separately (cortical bone: E_cort_ = 16 GPa, ν_cort_ = 0.36; trabecular bone: E_trab_ = 1 GPa, ν_trab_ = 0.3 [41]), as listed in Table 3 and shown in Figure 2A. The geometries were meshed with 10-node tetrahedral elements with quadratic functions (C3D10), which are well-suited for complex biological geometries such as the proximal femur and bending-dominated deformation fields [42,43]. A mesh convergence study was conducted following Oefner et al. [6], refining the mesh at least three times with a node increase ratio of 1:1.3–1:1.5 and a convergence criterion of 1–5%. This was performed for Bone 4 as a representative femur model (Table 1). Element shape quality was assessed using aspect ratios, with values between 1 and 4 being recommended for tetrahedral elements and the percentage of elements exceeding 3 being suggested to remain below 5% [43,44]. Each femur was then fully constrained 70 mm along the y-axis above the condyles, representing the embedding of the bone (Figure 2C). A load of 2000 N was applied via a reference point along the y-axis of the femoral coordinate system, kinematically coupled to a spherical surface region at the proximal aspect of the femoral head and extending 10 mm into the surface (Figure 2C). This magnitude was chosen to match the loading conditions of the round-robin study by Kluess et al. [9] and approximates the hip joint contact force during one-leg stance, which is reported as 238% of body weight [45]. Muscle forces were not included, consistent with simplified loading conditions commonly used in femoral FE benchmarking studies [9,33].

In addition to this baseline model, a sensitivity analysis was performed to assess the influence of material property assumptions, applying two variations (Case A and Case B) specified in Table 3.

To evaluate the results, the maximum horizontal and vertical displacements at the reference point coupled to the proximal aspect of the femoral head were calculated. In addition, the logarithmic strain values from ROI 1 and ROI 2, where the virtual strain gauges are located, were extracted and averaged. Von Mises stress was additionally calculated as a secondary parameter for the FE analysis, as it cannot be directly validated against the experiments, and it is therefore reported in Appendix A. The present FE analysis was developed in accordance with the experimental and computational work of Kluess et al. [9] to assess the feasibility of 3D ZTEMRI for subject-specific FE modeling. Accordingly, all findings were compared with those of the round-robin study by Kluess et al. [9]. In addition, the findings were discussed in the context of other relevant studies [33,34,35,36,37,38,39]. The FE analysis was performed on a conventional desktop computer (Windows 11 Pro, Processor: 12th Gen Intel^®^ Core^TM^ i5-12400, Intel Corporation, Santa Clara, CA, USA, CPU @ 2.50 GHz, 32 GB RAM).

## 3. Results

**Figure 3:**
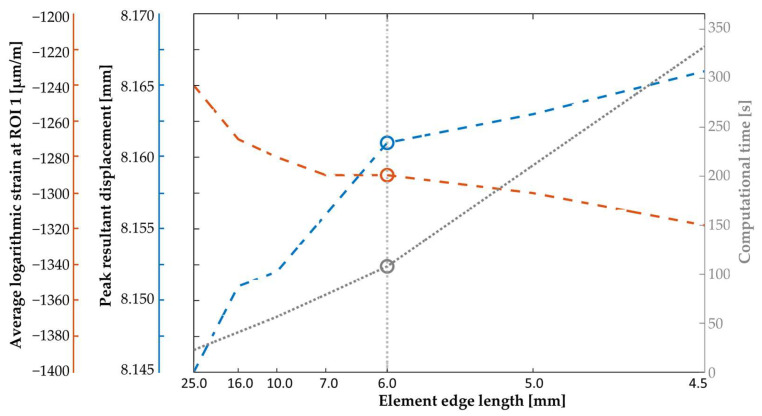
Convergence study analyzing the average strain in the region of interest (ROI) 1 and the peak resultant displacement for Bone 4 in comparison to the computational time. The circles indicate the chosen element size of 6 mm.

Four FE models of the femoral bone, generated from 3D ZTE MRI, were successfully analyzed. The mesh convergence analysis for Bone 4 (Figure 3) yielded an optimal global element edge length of 6 mm. The virtual strain gauges were meshed more finely with an element length of 1 mm. The final meshes achieved a mean aspect ratio of 1.63 with worst-case values below 5, and only 0.12% of elements exceeded an aspect ratio of 3, indicating good overall mesh quality. On average, each model consisted of 76,936 ± 4529 quadratic tetrahedral elements (C3D10) and required a 127 ± 11 s runtime.

**Figure 4:**
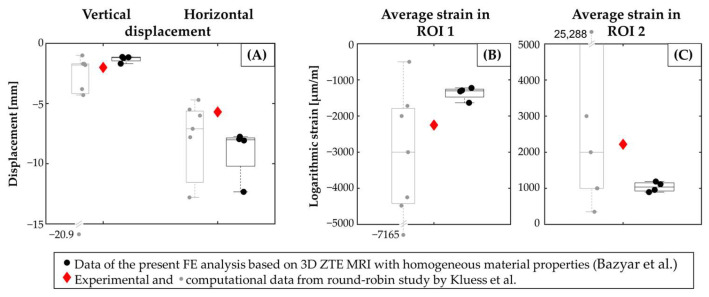
Predicted displacement and strain values in the present FE analysis based on 3D ZTE MRI data in comparison to the data derived in the round-robin study by Kluess et al. [9]. Homogeneous material properties for cortical and trabecular bone were employed based on Bazyar et al. [41]. (**A**) Maximum femoral head displacement in the vertical and horizontal directions. (**B**) Average strain at the inferior aspect of the femoral neck (ROI 1). (**C**) Average strain at the superior aspect of the femoral neck (ROI 2). Black boxplots and individual data points depict the results obtained in the present FE analyses. Gray boxplots with individual gray data points represent the results of the round-robin FE analysis reported by [9]. The red diamond indicates the corresponding experimental reference value from [9].

The results were largely consistent across the four FE models with respect to the maximum displacements of the femoral head and the average strains in the ROIs. After the application of 2000 N, the mean vertical displacement amounted to −1.30 ± 0.26 mm, while the horizontal displacement averaged −9.03 ± 2.21 mm. Regarding the strain distribution, the inferior aspect of the femoral neck (ROI 1) exhibited compressive strains of −1366.03 ± 182.70 µm/m, whereas the superior aspect (ROI 2) showed tensile strains of 1038.69 ± 135.82 µm/m. Figure 4 summarizes the predicted displacement and strain values of this FE study, as well as the experimental and computational reference values from the round robin study by Kluess et al. [9]. Here, the predicted maximum vertical and horizontal displacements, as well as the average strains in ROI 1 and ROI 2, deviate, on average, by 35%, 55%, 39%, and 52%, respectively, from the corresponding experimental measurements reported by Kluess et al. [9], yielding an overall mean deviation of approximately 46%.

**Figure 5:**
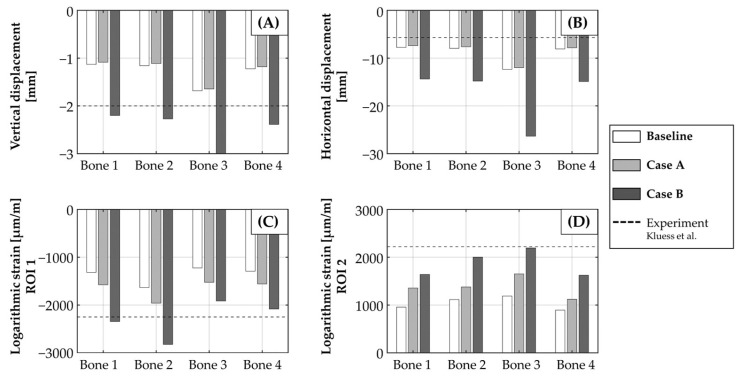
Sensitivity analysis of material properties and comparison with experimental reference data [9]. (**A**) Maximum vertical femoral head displacement and (**B**) maximum horizontal femoral head displacement. (**C**) Average strain in the inferior region of the femoral neck (ROI 1) and (**D**) average strain in the superior region (ROI 2) of the femoral neck. Results are shown for the baseline model (white) as well as for Case A (light gray) and Case B (dark gray) of the sensitivity analysis. The experimental reference values reported by [9] are included for comparison.

**Figure A1:**
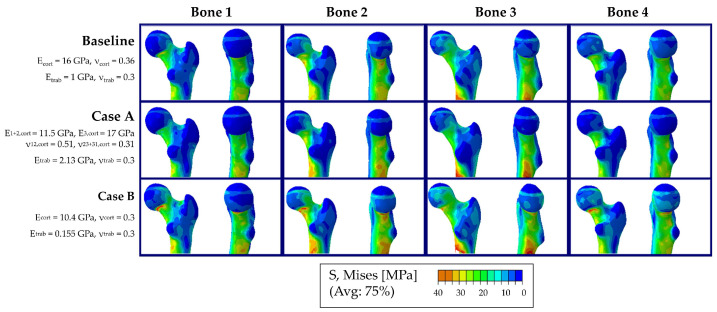
Distribution of von Mises stress in the femoral heads of Bones 1–4 (columns), shown as a secondary parameter to complement the strain and displacement results presented in the main text. Rows show the baseline model and two additional cases (Cases A and B) from the sensitivity analysis, which differ in the assigned material properties [41,46,47]. Across bones, Bone 3 consistently shows the highest von Mises stress values, while across cases, the material model used in Case B yields the highest von Mises stress values.

The sensitivity analysis investigated the influence of varying material properties on the FE results, focusing primarily on strain and displacement (Figure 5), while corresponding von Mises stress distributions are provided as a secondary parameter in Appendix A (Figure A1). The use of Case A resulted, on average, in a change of −3% or −0.04 mm in vertical displacement and −4% or −0.32 mm in horizontal displacement compared to the baseline model. Furthermore, the average logarithmic strain in the investigated regions changed by 21% or −288.34 µm/m in ROI 1 and 32% or 337.13 µm/m in ROI 2. When applying the material properties of Case B, larger deviations compared to the baseline model are present, with a mean variation of 106% or 1.42 mm in vertical displacement, 92% or 8.57 mm in horizontal displacement and 67% or −925.24 µm/m and 79% or 826.25 µm/m in the average logarithmic strains of ROI 1 and ROI 2, respectively. The comparison of the sensitivity analysis results with the experimental data from Kluess et al. [9] (Figure 5) showed an overall mean deviation of 38% for Case A (vertical displacement: 37%, horizontal displacement: 53%, strain ROI 1: 26%, strain ROI 2: 37%), and 65% for Case B (vertical displacement: 36%, horizontal displacement: 208%, strain ROI 1: 2%, strain ROI 2: 15%).

## 4. Discussion

Our present study investigated whether medical images from 3D ZTE MRI, acquired directly from human subjects, can serve as a reliable and radiation-free alternative to conventional CT for generating subject-specific FE models with sufficient geometric accuracy and anatomical fidelity. The results indicate that 3D ZTE MRI can differentiate cortical and trabecular regions and provide bone geometries suitable for FE model generation, while avoiding ionizing radiation. The FE models derived from these medical images showed biomechanical responses consistent with values reported in previous studies [9,33,34,35,36,37,38,39]. This suggests that 3D ZTE MRI may support accurate subject-specific biomechanical simulations. Beyond FE modeling, this approach also benefits other subject-specific computational workflows in biomechanics. These include musculoskeletal multibody models, which require accurate bone geometry, muscle attachment sites, and muscle parameters. The ability of MRI to capture both bone geometry and surrounding soft tissues, including muscle attachment sites, in a single examination can, therefore, simplify future model generation and improve anatomical representation.

### 4.1. Comparison with the Reference Round-Robin Study

The results of the present FE study were compared with those of Kluess et al. [9], who conducted an experimental axial-loading study on the femur and compared the results in a round-robin approach with FE analyses performed across several laboratories. Their FE models were built from the same specimen information, and six out of seven laboratories applied heterogeneous material properties derived from CT data. As grayscale values in 3D ZTE MRI do not directly correlate with Hounsfield units from CT images [48] and, therefore, heterogeneous material assignment is not feasible with 3D ZTE MRI, we used manual assignment into cortical and trabecular regions based on imaging data, which remains common practice in FE analyses [38,49]. This simplification substantially reduces computational time, facilitating rapid FE analysis with an average runtime of around 2 min, but might also influence the biomechanical behavior of the bone [4,6]. Although direct numerical comparison between our present study and the round-robin study is limited by differences in specimen geometry, the variability of our results is comparable to that reported in the round-robin study [9], which exhibited overall deviations between experimental and numerical results ranging from 22% to 354% (Figure 4). Kluess et al. [9] note that differences in results can likely be attributed to varying elasticity-density relationships, which have been shown to have a substantial influence on strain calculations [32], and to mapping strategies, which also influence numerical results [50,51].

The influence of the chosen bone material model becomes evident when comparing the results of the sensitivity analysis of the present study with the experimental data from Kluess et al. [9] (Figure 5). For Case A, displacement results were similar to the baseline model, while strain values were slightly higher, bringing them closer to the experimental reference data (strain ROI 1: 26%, strain ROI 2: 37%) and yielding the overall best agreement among the investigated cases with an overall deviation of 38%; this case differs from the baseline through an orthotropic cortical material definition with a higher Young’s modulus in the z-direction. Case B shows even better agreement with the experimental [9] strain values (strain ROI 1: 2%, strain ROI 2: 15%) but substantially overestimates horizontal displacement with 208% deviation, likely due to the comparatively low Young’s modulus assigned to cortical and trabecular bone. The horizontal displacement is overestimated across all case studies in the sensitivity analysis, whereas vertical displacement shows both over- and underestimation depending on the material model. This trend is similarly observed across the laboratories in the round-robin study [9]. Consequently, this highlights that material properties can substantially alter FE results. Especially when using novel medical image acquisition methods such as 3D ZTE MRI, clear reporting of model assumptions and sensitivity analyses is essential to assess how uncertain input parameters influence the results [6,9,51].

### 4.2. Comparison with Literature Data

In addition, several previous studies have investigated femoral loading scenarios experimentally and numerically. Experimental results under an axial load of 2000 N were, e.g., reported by Schileo et al. [35], Miura et al. [39] and Youssefian et al. [36], who reported vertical displacements of −3.2 mm, −2.5 mm and −1.5 mm, respectively. Keyak et al. [33] conducted experimental tests on 18 femoral specimens to develop CT-based heterogeneous material models; however, unlike our setup, the load was applied in the coronal plane at 20° to the shaft. Their experiments yielded a displacement of −1.5 mm at 2000 N, whereas their corresponding FE model predicted a displacement of −0.2 mm using subject-specific heterogeneous, isotropic properties. Similarly small numerical displacements were reported by Miura et al. [39], who demonstrated that the predicted response strongly depends on the chosen elasticity–density relationship: using the formulation of Keyak et al. [52], 2000 N resulted in a −0.2 mm displacement; that of Keller et al. [53] led to −1 mm, while the relationship proposed by Carter and Hayes [54] predicted failure below 2000 N. With a mean value for the experimental vertical displacements reported in four studies [33,35,36,39] of −2.19 ± 0.83 mm, the baseline model’s mean vertical displacement of −1.30 mm falls 40% below this experimental mean; however, this is consistent with the numerical predictions reported in the literature (−0.2 to −1 mm), which similarly yield smaller displacements [33,39].

Comparing strain values with those reported in the literature is challenging, as different regions of interest and loading scenarios are studied. Levadnyi et al. [34] performed numerical and experimental studies using intact femora at 3000 N loading and reported a minimum principal strain of around −1200 µm/m experimentally and −1700 µm/m numerically for the inferior side of the femoral head. In the same region, Polgar et al. [38] report numerically simulated principal strains for the muscle-standardized femur [37] of around −700 µm/m. With a literature mean of approximately −1200 ± 500 µm/m [34,38] across these studies, the baseline model’s mean strain of −1366 µm/m in the inferior region deviates by approximately 14%, remaining within the reported range.

Overall, the predicted strain and displacement results in the present study are consistent with both numerical and experimental results from previous studies, highlighting the feasibility of using 3D ZTE MRI for subject-specific FE models [8].

### 4.3. Limitations and Future Works

This feasibility study has several limitations. First, the study is limited to four female adults under 35 years old and does not account for sex-, age-, or ethnicity-specific differences in femoral bone geometry and material properties [55,56,57], limiting the generalizability of the findings. Second, the direct comparison between 3D ZTE MRI- and CT-derived bone geometries and FE models remains pending. Such a comparison was not feasible here, as the femoral specimen used in the study by Kluess et al. [9] was no longer available, and the present study relied on MRI images of healthy living subjects, for whom CT imaging was not ethically justifiable. This also means that the geometric accuracy cannot be guaranteed, and the exact placement of the virtual strain gauges cannot be precisely replicated due to inter-subject anatomical differences in bone geometry [58]. Future work should quantify differences in prediction accuracy and anatomical representation by directly comparing FE models derived from 3D ZTE MRI and CT. Third, subject specificity in the present FE models is currently limited to the reconstructed geometry, as individual variations in bone density, porosity, and microstructure, which are known to influence biomechanical behavior [35,50,59], are not captured by the simplified, literature-derived, homogeneous material assumptions applied. As there is no direct, well-established relationship between the grayscale values of 3D ZTE MRI data and bone’s mechanical properties, unlike CT imaging, where Hounsfield Units enable empirical density conversion via established elasticity-density relationships [35,52], a heterogeneous, density-based material model could not be implemented in the present study. The neglect of the medullary canal disregards heterogeneous stiffness distributions that contribute to local deformation differences, particularly in regions of high stress concentration [50]. Future studies should explore multi-material or gradient-material models derived from quantitative MRI mapping, for example, using UTE or ZTE sequences, and subsequently address their implementation, pending the development of reliable MRI-to-density calibration methods.

## 5. Conclusions

In conclusion, the present study demonstrates the feasibility of radiation-free 3D ZTE MRI for the development of subject-specific FE models of human femoral bone. The results indicate that 3D ZTE MRI can differentiate cortical and trabecular regions for FE model generation without ionizing radiation. The displacement and strain results fall within an acceptable and plausible range when compared with those from biomechanical experiments and in CT-derived FE models from a previous round-robin study and other previous studies, supporting the preliminary feasibility of this approach. The sensitivity analysis and comparisons with the literature highlighted the considerable influence of the assigned material properties on the model outputs, underscoring the need for refined MRI-based material assignment strategies. Direct validation against CT-derived models and experimental benchmarks remains necessary and represents an essential direction for future work. As 3D ZTE MRI avoids ionizing radiation, it holds the potential to enable studies involving healthy participants and thereby support the establishment of reference biomechanical datasets. Moreover, CT-like MRI approaches can enable simultaneous assessment of bone morphology and surrounding soft tissues, including muscle attachment sites, within a single examination.

## Figures and Tables

**Table 1:** Characteristics of participants (*n* = 4) recruited for this study.

Bone	Sex	Age	Height [cm]	Weight [kg]
1	Female	30	178	71
2	Female	24	173	72
3	Female	35	174	66
4	Female	31	178	65

**Table 2:** Magnetic resonance imaging parameters.

	3D ZTE MRI Coronal
Repetition time [ms]	674
Echo time [ms]	0.016
Field of view [mm]	350
Matrix [pixel]	280 × 280
Slice thickness [mm]	1.3
Voxel size [mm^3^]	1.3 × 1.3 × 1.3
Number of excitations	4
Bandwidth/pixel [Hz]	195
Flip angle [°]	1
Echo train length [-]	1
Acquisition time [s]	272

**Table 3:** Material properties used for the baseline model and the sensitivity analysis.

Case		Elastic Modulus [GPa]	Poisson’s Ratio	Literature
Baseline	Cortical	E_cort_ = 16	ν_cort_ = 0.36	Bazyar et al. (2023) [41]
	Trabecular	E_trab_ = 1	ν_trab_ = 0.3	
				
Case A	Cortical	E_1,cort_ = E_2,cort_ = 11.5, E_3,cort_ = 17	ν_12,cort_ = 0.51, ν_23,cort_ = ν_31,cort_ = 0.31	Bori et al. (2022) [46]
	Trabecular	E_trab_ = 2.13	ν_trab_ = 0.3	
Case B	Cortical	E_cort_ = 10.4	ν_cort_ = 0.3	Marco et al. (2018) [47]
	Trabecular	E_trab_ = 0.155	ν_trab_ = 0.3	

## Data Availability

The original contributions presented in this study are included in the article/Appendix A. Further inquiries can be directed to the corresponding author.

## Abbreviations

The following abbreviations are used in this manuscript:

FE	Finite element
CT	Computed tomography
MRI	Magnetic resonance imaging
UTE	Ultrashort echo time
ZTE	Zero-echo-time
ROI	Region of interest
